# Antifungal Peptides as Therapeutic Agents

**DOI:** 10.3389/fcimb.2020.00105

**Published:** 2020-03-17

**Authors:** Miguel Fernández de Ullivarri, Sara Arbulu, Enriqueta Garcia-Gutierrez, Paul D. Cotter

**Affiliations:** ^1^APC Microbiome Ireland, University College Cork, Cork, Ireland; ^2^Food Bioscience Department, Teagasc Food Research Centre, Fermoy, Ireland; ^3^Gut Microbes and Health, Quadram Institute Bioscience, Norwich, United Kingdom

**Keywords:** antifungal peptides, antimicrobial peptides, mycoses, antimicrobial resistance, production, new therapies

## Abstract

Fungi have been used since ancient times in food and beverage-making processes and, more recently, have been harnessed for the production of antibiotics and in processes of relevance to the bioeconomy. Moreover, they are starting to gain attention as a key component of the human microbiome. However, fungi are also responsible for human infections. The incidence of community-acquired and nosocomial fungal infections has increased considerably in recent decades. Antibiotic resistance development, the increasing number of immunodeficiency- and/or immunosuppression-related diseases and limited therapeutic options available are triggering the search for novel alternatives. These new antifungals should be less toxic for the host, with targeted or broader antimicrobial spectra (for diseases of known and unknown etiology, respectively) and modes of actions that limit the potential for the emergence of resistance among pathogenic fungi. Given these criteria, antimicrobial peptides with antifungal properties, i.e., antifungal peptides (AFPs), have emerged as powerful candidates due to their efficacy and high selectivity. In this review, we provide an overview of the bioactivity and classification of AFPs (natural and synthetic) as well as their mode of action and advantages over current antifungal drugs. Additionally, natural, heterologous and synthetic production of AFPs with a view to greater levels of exploitation is discussed. Finally, we evaluate the current and potential applications of these peptides, along with the future challenges relating to antifungal treatments.

## Introduction

Fungi are extraordinary, ubiquitous organisms that play critical roles in complex ecosystems. These eukaryotes range from giant forms to microscopic unicellular molds and yeasts. In recent years fungi have been recognized as an integral part of our commensal microbiota at different body sites (e.g., gut, oral cavity, skin, lung, vagina), although there is no consensus on what constitutes the standard mycobiome composition (Huffnagle and Noverr, 2013; Enaud et al., 2018; Kapitan et al., 2018) and some studies point out that gut fungi may come from oral and dietary sources (Auchtung et al., 2018). Indeed, fungi have been used as a source of food and for food processing for thousands of years (Campbell-Platt and Cook, 2008). Fungi are also routinely employed in many industrial processes including the production of peptides, enzymes, vitamins, organic acids, and antibiotics (Money, 2016; Mukherjee et al., 2018).

However, fungal infections, or mycoses, have become a serious threat to human health, causing a wide range of infections in humans. It is estimated that fungal diseases affect more than one billion people globally, of whom 150 million suffer from severe infections (Bongomin et al., 2017). These range from superficial and subcutaneous life quality-debilitating infections affecting the skin, keratinous tissues and mucous membranes (Kaushik et al., 2015), to systemic infections that can be life-threatening involving the brain, heart, lungs, liver, spleen, and kidneys (Rautemaa-Richardson and Richardson, 2017). The latter are especially worrying in the case of immunocompromised patients with HIV/AIDS or autoimmune diseases, and in those undergoing anticancer chemotherapy or organ transplantation.

The main human fungal pathogens are *Candida albicans, Cryptococcus neoformans*, and *Aspergillus fumigatus*, but, worryingly, non-albicans *Candida* spp. such as *C. auris*, in addition to other infectious agents such as *Histoplasma capsulatum* or *Malassezia furfur* are emerging. For a comprehensive review on main fungal pathogens affecting humans see (Roemer and Krysan, 2014).

Four major classes of antifungal agents dominate the market: azoles, which inhibit the synthesis of ergosterol; polyenes, which interact with fungal membrane sterols physicochemically; echinocandins that inhibit glucan synthesis; and fluorinated pyrimidines, which interfere with pyrimidine metabolism, leading to the inhibition of DNA and RNA biosynthesis (Roemer and Krysan, 2014). However, the high mortality of invasive fungal infections, the long course of treatments required, narrow spectrum activity and cross-resistance due to similar mechanisms of action across drugs has triggered the search for safer alternatives with reduced toxicity or other enhanced features. As eukaryotes, a particularly great challenge is to identify pathogen-specific targets not present in human cells.

Monoclonal antibodies, cytokine immunotherapy, vaccines and antimicrobial peptides (AMPs) have emerged as new biopharmaceuticals to prevent or treat fungal infections (Nicola et al., 2019). There is an increasing interest in peptides as promising novel antibiotic agents. Peptides can mimic natural ligands and therefore function as agonists or antagonists. Regarding their use as drugs, peptides are highly selective, effective and well-tolerated (Fosgerau and Hoffmann, 2015). Among the broader peptide category of antimicrobials, AMPs are gene-encoded conserved molecules produced by all organisms, from bacteria to humans. Compared with conventional antibiotics, which are generally targeted against bacteria or fungi, AMPs can exhibit broad antimicrobial activity including bacteria, fungi, parasites, viruses, protozoa and even some cancer cells (Hancock and Chapple, 1999). Being effective against this broad range of targets might imply different modes of action and prevent bacteria and fungi from developing resistance. AMPs produced by higher organisms are involved in the innate and secondary immune responses against microbes, while those produced by bacteria frequently kill other bacteria competing for the same ecological niche (Zhang and Gallo, 2016). AMPs also confer protection by contributing to gut homeostasis, and modulation of host inflammatory responses. Notably, AMPs with a narrow antimicrobial spectrum have particularly great therapeutic potential as they are less likely to cause disruption of the host microbiota.

**Figure 1:**
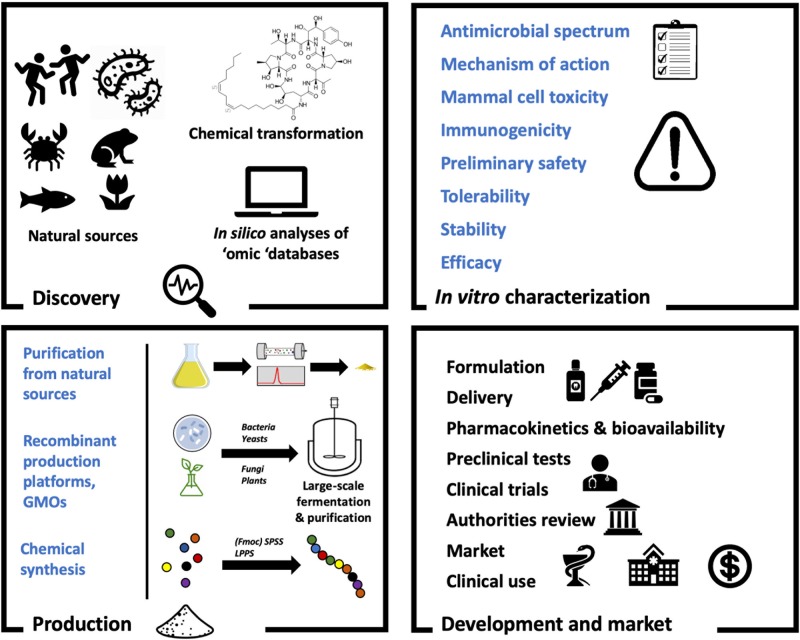
The antifungal peptide development process. As with any drugs, AFPs must undergo several stages of development to reach clinical use. When the candidate molecule shows promise as a therapeutic (Discovery) it must be characterized (*In vitro* characterization). In order to facilitate this, sufficient amounts of the peptide must be available (Production). Finally, the molecule will be subjected to formulation processes and preclinical tests before going into clinical trials and receive approval (Development and market).

In this review we provide an overview of the bioactivity and classification of AMPs with antifungal activity, known as antifungal peptides (AFPs), as well as their mode of action and advantages over current antifungal drugs. Additionally, natural, heterologous and synthetic production of AFPs with a view to greater levels of exploitation is discussed. In this regard, Figure 1 shows a general overview on AFP development. Finally, we evaluate the current and potential applications of these peptides, together with future challenges in relation to antifungal treatments.

## Types of Antifungal Peptides and Bioactivity

As of November 2019, there were 1,133 peptides with antifungal properties reported in the Antimicrobial Peptide Database (APD3) (Wang et al., 2016). AFPs have been classified following a number of different criteria, such as structure or mode of action. However, the most accepted classification is based on the peptide origin: natural, semisynthetic or synthetic (De Lucca, 2000). Here, we summarize some of the most important features of natural peptides and we describe how synthetic AFPs are designed.

### Natural Peptides

Natural AFPs are produced by a number of different species of Bacteria, Archaea, and Eukarya isolated from natural sources (De Lucca and Walsh, 1999). Most natural AFPs have been discovered by testing their antagonistic activity *in vitro* against pathogenic fungi (Mania et al., 2010; Freitas and Franco, 2016; McNair et al., 2018). However, with the rise of sequencing technologies and the drop in associated costs, new strategies for prediction and discovery of new AFPs are emerging. New methods such as template-based, docking simulations, hidden Markov model and other sequence-based methods allow for novel *in silico* prediction of AFPs (Schneider and Fechner, 2005; Fjell et al., 2007, 2008; Robinson, 2011; Garrigues et al., 2017; Agrawal et al., 2018).

Natural AFPs are grouped in families according to their origin (Table 1). Those produced by bacteria and fungi are arguably of greatest interest with respect to medical applications (Essig et al., 2014). Although some other sources, such as plants, are a rich source of AFPs, they are mainly employed for other purposes, such as the control of phytopathogens, and thus, they are not further discussed in this review (De Lucca, 2000). Notably, the first archaeal AMP with antifungal and anti-biofilm capabilities against clinical fungal pathogens was recently reported (Roscetto et al., 2018).

**Table 1:** Natural AFPs families with clinical applications.

**Origin**	**Family name**	**Chemical characteristics**	**Mode of action**	**Active against**	**Examples**	**References**
Archaea	Cryptic CAMP-like	Cationic	Targets cell wall (still being investigated)	*Candida* spp.	VLL-28	Roscetto et al., 2018
Bacteria	Iturin	Small cyclic peptidolipids with a lipid-soluble β-amino acid linked to a peptide with D- and L- amino acids.	Lysis by pore formation in membranes	*niger* *albicans* *F. oxysporum*	Iturin A Bacillomycin F Bacillomycin L	Landy et al., 1948; De Lucca and Walsh, 1999; De Lucca, 2000
	Syringomycins	Small cyclic lipodepsipeptides	Forms voltage-sensitive ion channels Alters protein phosphorylation and H^+^-ATPase activity Pore formation	*Candida* spp. *Cryptococcus* spp. *Aspergillus* spp.	Syringomycin-E (SE) Syringostatin A Syringotoxin B	Sinden et al., 1971; De Lucca and Walsh, 1999; De Lucca, 2000
Fungi	Nikkomycins	Peptide nucleosides	Inhibit chitin biosynthesis	*Blastomyces dermatitidis, C. albicans*	Nikkomycin X, Z	McCarthy et al., 1985; Hector et al., 1990; De Lucca and Walsh, 1999; De Lucca, 2000
	Polyoxins	Peptide nucleosides	Inhibit chitin biosynthesis	*C. albicans*	Polyoxin A, B, D	Suzuki et al., 1965; Isono et al., 1969; Hori et al., 1974; De Lucca and Walsh, 1999; De Lucca, 2000
	Echinocandins	Cyclic hexapeptides with N-linked acyl lipid side chains	Inhibit glucan synthesis	*Candida* spp. *Aspergillus* spp.	Echinocandins, pneumocandins, aculeacins, mulundocandins, WF11899	De Lucca and Walsh, 1999; De Lucca, 2000; Eschenauer et al., 2007
	Aureobasidins	Cyclic depsipeptide	Lysis by altering actin assembly and delocalizing chitin in fungal walls / sphingolipid synthesis inhibition	*B. dermatitidis* *Candida* spp.	Aureobasidin A	Endo et al., 1997; De Lucca, 2000
	Leucinostatins	Contains five unusual amino acids, 4-methylproline (MePro), 2-amino-6-hydroxy-4-methyl- 8-oxodecanoic acid (AHMOD), threo-β-hydroxyleucine (HyLeu), three 2-aminoisobutyric acid (Aib), and β-alanine (b-Ala)	Uncouplers mitochondria	*Candida* spp.	Leucinostatins A, B, D, H and K	De Lucca and Walsh, 1999; De Lucca, 2000; Abe et al., 2018
Amphibians	Magainins	Helical, amphiphilic	Lysis by dissipating ion gradient in cell membranes	*C. albicans*	Magainin 2	De Lucca and Walsh, 1999; De Lucca, 2000
	Dermaseptins	Linear, cationic, lysine-rich	Lysis by interacting with lipid bilayers	*flavus* *fumigatus* *F. oxysporum*	Dermaseptin	Mor et al., 1991; De Lucca and Walsh, 1999; De Lucca, 2000
	Skin-PYY	Similar to neuropeptide NPY and gastrointestinal peptide PYY, C- terminal α-helix domain conserved	Membrane disruption	*C. neoformans* *C. albicans* *A. fumigatus*	Skin-PYY	Vouldoukis et al., 1996; De Lucca, 2000
Plants	Defensins	Small, highly stable, cysteine-rich peptides	Membrane pore formation (carpet or toroidal pore), ion efflux, induction of reactive oxygen species and programmed cell death.	*C. albicans, C. krusei, A. flavus and Fusarium solani*	RsAFP1, RsAFP2, SPE10, NaD1	Bondaryk et al., 2017; Sher Khan et al., 2019
Insects	cecropins	Linear	Cell lysis	*A. fumigatus*	Cecropins A and B	De Lucca et al., 1998; Bondaryk et al., 2017
	Cysteine-rich peptides	Hairpin-like beta-sheet structure	Cell lysis	*C. albicans*	Defensins, Drosomycin, thanatin	Dimarcq et al., 1998; Bondaryk et al., 2017
Peptides from aquatic sources	Aciculitins	Cyclic peptides and lipid residues	Cell lysis	*C. albicans*	Aciculitins A-C	Bewley and Faulkner, 1994; Bondaryk et al., 2017
	Theonegramide	Glycopeptide with unusual amino acids	Unknown	*C. albicans*	Theonegramide	Bewley and Faulkner, 1994; Bondaryk et al., 2017
	Laxaphycins	Cyclic peptides	Unknown	*C. albicans*	Laxaphycins A, B, D and E	Bondaryk et al., 2017
	Defensins	β-sheet	Chitin binding	*C. albicans*	Tachycitin, “big defensin”	Bondaryk et al., 2017
Mammalian	α-defensins	β-sheet with cysteines forming intramolecular disulphide bonds	Cell lysis	*C. albicans* *C. neoformans*	HNP-1, HNP-2, HNP-3, NP-1, NP-2, NP-3, NP-4	De Lucca and Walsh, 1999; De Lucca, 2000
	β-defensins	β-sheet with cysteines with a disulphide motif different from α-defensins. Amino termini are blocked with a pyroglutamyl residue	Cell lysis	*C. albicans*	Tracheal antimicrobial protein (TAP) Gallinacins−1,−1α, 2	De Lucca and Walsh, 1999; De Lucca, 2000
	Protegrins Cathelicidins	Cationic, cysteine-rich β-defensins	Pore formations and lysis	*C. albicans*	Protegrins 1, 2 and 3	De Lucca and Walsh, 1999; De Lucca, 2000; Bondaryk et al., 2017
	Histatins	Basic and neutral helical peptides	Induction of cell death, osmosis stress	*C. albicans*	Histatins 1, 3, 5	Koshlukova et al., 1999; De Lucca, 2000; Bondaryk et al., 2017

Typically, natural AFPs adopt an α-helix structure, β-hairpin or sheet (containing two cysteine residues) or mixed α-helix/β-sheet structures upon interaction with membranes. Some of these peptides are rich in specific amino acids and, as a result, are classified as glycine-rich, proline-rich, arginine-rich, histidine-rich, and tryptophan-rich (Bondaryk et al., 2017). However, the structure of most AFPs has not yet been determined.

Posttranslational modifications also play a major role in the final three-dimensional structure and bioactivity of AMPs but, unfortunately, cannot be predicted with current *in silico* tools (Agrawal et al., 2018). The most common posttranslational modifications in natural AFPs involve glycosylation or the addition of carbohydrates, normally observed in asparagine, or serine/threonine residues (Guo et al., 2012; Bednarska et al., 2017). The latter has been shown to improve antifungal activity in some cases. Other modifications include halogenation such as chlorination (Andreu and Rivas, 1998; Shinnar et al., 2003), phosphorylation, which can increase stability (McDonald et al., 2011) or hydroxylation, mainly observed at lysine, arginine, tryptophan, and phenylalanine residues with differing effects on antifungal activity (Houwaart et al., 2014; Akkam, 2016). Finally, cyclization of AMPs, which is not considered a posttranslational modification, improves antimicrobial activity in general, reduces toxicity and improves stability against proteases (Akkam, 2016).

### Semisynthetic and Synthetic Peptides and Structure Activity Relationships (SAR) Criteria for Their Design

Antimicrobial semisynthetic and synthetic peptides are generally made with a view to improving pharmacological properties, reducing side effects and/or lowering the immunogenicity of natural peptides. Pharmaceutical formulation will also help to enhance their stability and bioavailability. As an example of synthetic transformation, it was found that the hemolytic activity of the natural echinocandin B AFP was significantly reduced by the replacement of the linoleoyl side-chain with either octyloxybenzoyl (cilofungin) or pentyloxyterphenyl (anidulafungin) side chains (Emri et al., 2013).

Structure-activity relationships (SAR) are a key element used for the design and development of synthetic peptides (Lum et al., 2015). There are several biophysical properties that can determine antifungal activity, such as net charge, stereospecificity, hydrophobicity, amphipathicity, secondary structure and peptide length, with some of these characteristics being interdependent. These properties have been extensively reviewed previously (Akkam, 2016). Most AFPs are cationic, but neutral and anionic AFPs have also been described. When present, cationic charges play a role in the electrostatic binding of AMPs to negatively charged membranes. Therefore, an increase in the positive net charge beyond a threshold might lead to a stronger activity on the membrane. However, positive charges are not a prerequisite for antimicrobial activity, and anionic peptides interact with the membrane peptide through specific amino acid distributions (Yeaman and Yount, 2003; Lakshminarayanan et al., 2014).

Most AFPs are non-stereospecific (Akkam, 2016). In the same way, there is no dominant conformation among AFPs. Thus, the main differences between peptides come from sequence and secondary structure variation (Emri et al., 2013). Hydrophobicity and amphipathicity are essential factors for peptide membrane interactions and membrane permeabilization (Lum et al., 2015), and important variables in the design of synthetic peptides. Hydrophobicity is defined as the percentage of hydrophobic residues within a peptide, ranging between 30 and 60% for most AMPs. An increase in hydrophobicity and amphipathicity usually correlates with an increase in antifungal activity, but also with higher hemolytic activity. The presence of tryptophan is also linked to hemolytic activity since it interferes with lipid polymorphism in the membranes (Schibli et al., 2002).

The peptide length of AFPs is important for the secondary structure and mode of action. Most AFPs have 11–40 residues. It has been described that 7–8 amino acids are needed to form amphipathic structures in AMPs (Bahar and Ren, 2013), while <20 amino acids limit the ability of a peptide to form transmembrane structures in the fungal membrane (Rothstein et al., 2001; Akkam, 2016). Nevertheless, longer lengths may also affect cytotoxicity, stability and manufacturing costs. In order to overcome these hurdles, short antimicrobial peptides (SAMPs), containing 2–10 amino acids, are attracting attention as less toxic and more stable alternatives. Overall, SAMPs have simpler amino acid composition and they are easier to synthesize and modify chemically with a view to improving toxicity, stability, half-life or specificity. Moreover, they are also less immunogenic (Duncan and O'Neil, 2013; Fox, 2013).

Some of the semisynthetic and synthetic peptides studied with a view to clinical applications are summarized in Table 2. Considering all of the factors mentioned above, different strategies have been employed for synthetic peptide design. Combinatorial libraries generate model peptides suitable for SAR studies (Blondelle and Lohner, 2000). The template-based strategy, known as *de novo* design, uses peptides with known antifungal activity as scaffolds (Lum et al., 2015). As an example, Agrawal *et al*., performed *in silico* studies by machine learning techniques based on AFP and non-AFP sequences revealing a higher frequency of certain residues (C, G, H, K, R, and Y) and the prevalence of R, V, K at N-terminus and C and H at the C-terminus of peptides (Agrawal et al., 2018).

**Table 2:** Examples of semisynthetic and synthetic AFPs with clinical applications.

**Origin**	**Name**	**Structure**	**Mode of action**	**Active against**	**References**
Semisynthetic	Cilofungin (LY 121019)	Lipopeptide	Glucan synthesis	*C. albicans* *A. fumigatus*	Pfaller et al., 1989; De Lucca, 2000
	LY 303366	Lipopeptide	Glucan synthesis	*Candida* spp.	Karlowsky et al., 1997; De Lucca, 2000
	FK 463	Lipopeptide	Glucan synthesis	*Candida* spp. *Aspergillus* spp.	De Lucca, 2000; Mikamo et al., 2000
	L-693,989	Lipopeptide	Glucan synthesis	*C. albicans* *P. carinii*	Balkovec et al., 1992; De Lucca, 2000
	PMAP-23	α-helix	Membrane	*C. albicans*	Lee et al., 2002; Bondaryk et al., 2017
	KU2	α-helix	Membrane	*C. albicans*	Lum et al., 2015; Bondaryk et al., 2017
	KU3	α-helix	Membrane	*C. albicans*	Lum et al., 2015; Bondaryk et al., 2017
Synthetic	dF21-10K	Linear-kaxins	Membrane	*C. albicans* *C. tropicalis*	Burrows et al., 2006; Bondaryk et al., 2017
	KSL-W	α-helix decapeptide	Membrane	*C. albicans*	Semlali et al., 2011; Bondaryk et al., 2017
	B4010	Tetravalent dendron—Polylysine dendrons (PLL)	Membrane	*C. albicans*	Lakshminarayanan et al., 2014; Freitas and Franco, 2016; Bondaryk et al., 2017
	L1	Polyamidoamine (PAMAM) dendrimers	Intercalation with DNA	*Candida* spp.	Ottaviani et al., 2016; Bondaryk et al., 2017
	Killer peptide (KP)	Dimeric; β-sheet	Unknown	*Candida spp*. *Cryptococcus neoformans* *P. carinii Paracoccidioides brasiliensis* *A. fumigatus*	Magliani et al., 2011

Another strategy is to target virulence traits (Bondaryk et al., 2017). However, this approach is not efficient enough due to the expression of different virulence factors in fungi cells. For this reason, the generation of molecules with multiple ligands like dendrimers, or tree-like molecules, that have radially distributed layers of branches named generations, have been explored (Esfand and Tomalia, 2001).

## Models of Mechanisms of Action

Whereas some peptides have primarily antifungal activity, such as lipopeptides (e.g., echinocandins) or histidine-rich (e.g., the linear histatins or branched HK), membrane-disrupting peptides (e.g., magainins, protegrins) have a broader antimicrobial spectrum, including bacteria, fungi and viruses. Here, we summarize the hypothetical mechanisms of action of AFPs with activity against fungal pathogens.

### Peptides With Primarily Antifungal Properties

#### Inhibition of 1,3-β-Glucan Synthesis

β-glucan synthase is involved in cell wall integrity. Cyclic lipoproteins can non-competitively inhibit it, resulting in destabilization of the cell wall, leading to susceptibility to osmotic stresses and cell lysis. 1,3-β-glucans are involved in the division septum and assembly of the acropore wall as well; consequently, β-glucan synthase inhibitors affect these structures. β-glucan synthase is ubiquitous among fungi including *Candida, Aspergillus, Cryptococcus*, and *Pneumocystis* species. However, in mycelious fungi, it is found in tips of the growing hyphae, which makes them less sensitive. Inhibition of β-glucan synthase results in negative feedback, causing cell cycle arrest. Echinocandins and pneumocandins, aculeacins (A-D, F), mulundocandins and WF11899 (A, B, and C) as well as the killer toxin from *Williopsis mrakii*, WmKT are representative β-glucans synthase inhibitors (Guyard et al., 2002; Matejuk et al., 2010; van der Weerden et al., 2013).

#### Inhibition of Chitin Biosynthesis in the Cell Wall

Chitin, found in the fungal cell wall, is essential to maintain cell integrity and is absent in vertebrates. Aureobasidins are cyclic lipophilic 8-mer depsipeptides with an α-hydroxyacid that display two mechanisms of action: the disruption of cell wall/membranes by altering the assembly of actin and chitin (Endo et al., 1997) and the interruption of sphingolipids synthesis (Nagiec et al., 1997). Several members of the aureobasidin family exert anti-*Candida* activity. Nikkomycins are structural analogs of uridine diphosphate N-acetylglucosamine, a major constituent of chitin. These AFPs have been shown to inhibit the synthesis of chitin in *C. albicans* in both *in vitro* and *in vivo* studies (McCarthy et al., 1985) while human cells were not affected. They also show significant activity against *C. immitis, B. dermatitidis*, and moderate activity against *H. capsulatum* (Hector et al., 1990; Clemons and Stevens, 1997), but these agents are not active against filamentous fungi.

#### Selective Activity on Membranes

Rs-ARF2 is a 50 amino-acid residue plant defensin that has three-stranded β-sheets and an α-helix, structure that is stabilized by four disulfide bonds. Rs-ARF2 targets the fungus-specific membrane glucosylceramide inducing membrane permeability, which causes Ca^2+^ uptake, efflux of K^+^ and medium alkalinization. This defensin also induces the production of toxic reactive oxygen species intracellularly. Rs-AFP2 and analogs (with arginines replacing neutral amino acids) were found to inhibit *A. flavus* and *Fusarium solani, C. albicans* and *C. krusei*. *C. glabrata*, which does not contain this fungus-specific ceramide, is not inhibited. This peptide and its analogs (e.g., NaD1, Rs-ARF1, SPE10) have little cytotoxicity against mammalian cells at dosages that are inhibitory to fungal pathogens (Matejuk et al., 2010; Sher Khan et al., 2019).

Iturins, produced by *Bacillus subtilis*, are cyclic peptides with a lipophylic β-amino acid linked to a D and L amino acids, causing pore formation in membranes and leakage of key ions (Besson and Michel, 1984). Their antimicrobial activity is limited primarily to fungi, with little effect on bacteria. Unfortunately, iturins are toxic to mammalian cell membranes. In contrast to most antimicrobial peptides which are cationic, iturins can be anionic (bacillomycin L) or neutral (iturin A). One member of the family, bacillomycin F effectively inhibits *A. niger, C. albicans*, and *C. tropicalis*. Although this group of peptides is effective against dermatomycoses in humans and animals, they induce high levels of hemolysis (Latoud et al., 1986).

The mammalian peptide Histatin 5 contains seven histidines, four arginines, and three lysines which allow the peptide adopting an α-helical conformation in non-aquous environments (Raj et al., 1998). Histatin 5 binds to the Ssa2p, a 70-kDa cell wall protein required for the internalization of histatin 5 into cells (Li et al., 2006). The polyamine transporters Dur3 and Dur31 are necessary for the uptake of the peptide as well (Kumar et al., 2011), which needs to be translocated, not endocyted to exert an effect on *C. albicans* cells (Jang et al., 2010). If the cell is under respiratory metabolism, histatin 5 disrupts the integrity of mitochondrial membrane (Helmerhorst et al., 1999). Propidium iodide (PI) uptake and ATP release also occurs despite cell lysis does not appear to be induced by the peptide (Helmerhorst et al., 1999; Koshlukova et al., 1999). Subsequent ATP binding to surface P2X receptors induce signaling cascades leading to cell death.

### Broad Spectrum Antimicrobial Peptides

Most AMPs affect a number of organisms including bacteria, fungi, and envelope-containing viruses. The most representative non-specific AMPs can be grouped as linear peptides (e.g., cecropins, magainins, cathelicidins, bombinins, lactoferrin-derived peptides) or cyclic peptides (e.g., mammalian defensins, poultry gallinacins, macrocyclic peptides, syringomycins, tachystatins) (Matejuk et al., 2010). Membrane disruption by the formation of toroidal pore is common to most peptides, resulting in leakage of essential molecules and ions with general loss of membrane functionality. Additional mechanisms include membrane thinning, formation of non-bilayer intermediates by interchelation of peptide-membrane, formation of anionic lipid-peptide domains, lipid flip-flop, demixing and clustering, and alteration of membrane potential (Shai, 2002; Bechinger and Lohner, 2006; Huang, 2006; Melo et al., 2009; Nguyen et al., 2011; Rautenbach et al., 2016). In addition, some of the peptides may have other effects such as DNA damage, apoptosis induction, inhibition of DNA replication and RNA or protein synthesis.

For supplementary reviews on the mechanisms of action and classification of antimicrobial peptides see reviews by Matejuk (Matejuk et al., 2010), van der Weerden (van der Weerden et al., 2013), and Rautenbach (Rautenbach et al., 2016).

### Advantages of AFPs

Existing antifungal agents exhibit a diversity of drawbacks that reduce their efficiency as therapeutic tools against fungal infections. The existence of only a few approved classes of antifungal drugs and the increasing antifungal resistance further complicates the selection of an appropriate antifungal therapy (Sanguinetti et al., 2015; Pappas et al., 2018). Ergosterol synthesis, ergosterol in membranes and cell wall synthesis are the targets of azoles, polyenes and echinocandis, respectively. The low diversity of mechanisms of action of current treatments represents a problematic situation when fungal pathogens are multi-resistant to antifungals (Rautenbach et al., 2016). Furthermore, existing antifungal drugs are associated with adverse drug reactions such as hypokalemia, infusion reaction, nephrotoxicity, hepatotoxicity and gastrointestinal affections, among others (Chen et al., 2011; de Souza et al., 2016; Kyriakidis et al., 2017; Pappas et al., 2018). Some antifungal drugs affect common eukaryotic targets present both in pathogenic fungi and human cells (Rautenbach et al., 2016). This makes the development of novel efficient and non-toxic antifungal therapies more difficult than that of antibacterial ones.

AFPs have diverse advantages over the current antifungal drugs, which are directly related to their mechanisms of action and molecular targets. AFPs are particularly promising because they can recognize multiple microbial targets, thus reducing the possibility of resistance development (Rautenbach et al., 2016), a topic that is discussed in more detail in the following section. These microbial targets include fungal membranes, different cell wall components and molecules related to physiological processes, such as RNA, DNA and protein synthesis and cell cycle (van der Weerden et al., 2013; Bondaryk et al., 2017).

Regarding the absence of side effects, several AFPs target specific conserved fungal molecules, such as glucosylceramide, mannosyldiinositol phosphorylceramide, enzymes related to ergosterol or β-glucan synthesis, among others. This translates into high pathogen selectivity and reduces the probability of cytotoxicity against mammalian cells (Rautenbach et al., 2016). However, it does not ensure the absence of cytotoxicity (e.g., aculeacins, a type of echinocandin that targets the fungal 1,3-β-glucan synthase, displays hemolytic activity) (Matejuk et al., 2010). Over the last 13 years, three echinocandins, anidulafungin, caspofungin, and micafungin, have been approved in Europe and USA (Pound et al., 2011; Beyda et al., 2012). These peptides are poor substrates for cytochrome P450, which translates into both lower hepatotoxicity and pooled risk for discontinuation of treatment (3.7–4.8%) compared to other antifungals (Kyriakidis et al., 2017). The synthetic peptide killer peptide (KP) is another example that has demonstrated very promising antifungal activity without cytotoxicity against peripheral mononuclear blood cells *in vitro* or side effects in murine trials. This peptide seems to have a specific interaction with 1,3 β glucans and only the dimeric form of the peptide is active (Magliani et al., 2011).

In addition, AFPs show reduced cytotoxicity (Matejuk et al., 2010). Two reasons may explain this phenomenon. Firstly, there is a stronger interaction between the negatively charged fungal membrane (due to the higher content of phosphatidylinositol and phosphatidic acid) and the cationic charges of the peptides, in contrast to mammalian cell membranes, which are predominantly neutral to host mammals (due to the high content of phosphatidylcholine). Secondly, some AFPs target membrane lipids unique to fungi and absent from mammalian cells, which also reduces toxicity (Nguyen et al., 2011; Rautenbach et al., 2016).

Accumulating evidence demonstrates that the therapeutic activity of AMPs is multifactorial and not mediated only by their direct antimicrobial effect. Host defense peptides (HPDs), like defensins and cathelicidins families, often exert angiogeninc, immunomodulatory and anti-inflammatory effects and may also induce the recruitment of the adaptive immune response, which has been reported in several papers and reviews (Zasloff, 2002; Hirsch et al., 2008; Steinstraesser et al., 2009; Magliani et al., 2011; Hsieh and Hartshorn, 2016; Li et al., 2017).

### Resistance to AFPs

Membrane remodeling of C. albicans has been associated with its resistance to current non-peptide antifungal drugs, mainly ergosterol-sphingolipid-rich lipid rafts containing multi-drug resistance (MDR) proteins attached to the membrane (Mukhopadhyay et al., 2004; Pasrija et al., 2005, 2008; Shahi and Moye-Rowley, 2009). In the case of resistance to AMPs and AFPs it is important to note, as discussed previously, that these peptides frequently function through membrane interaction, but that additional modes of action for microbial inhibition have also been demonstrated (Wu et al., 1999). Microorganisms, such as fungi, evolve rapidly, and can adapt quickly when exposed to antibiotics and antifungal drugs. However, it is important to note that cell membranes are slower to evolve. The rapid and potent effect on membrane, coupled with other inhibitory mechanisms exhibited by AFPs, *de novo* resistance is less likely to emerge in target microorganisms (Yeung et al., 2011).

As is the case for currently used antimicrobial drugs, the overuse of AFPs could accelerate the occurrence of fungal resistance. This issue complicates the application of antifungal therapeutics. Indeed, extensive echinocandin usage in hospitals has led to an increase in the number of strains with acquired (secondary) resistance to these first-line antifungals, especially among strains of *C. glabrata* (Sanguinetti et al., 2015; Pappas et al., 2018). The potential for the emergence of high level resistance to AMPs has been debated, but it is likely to occur at a reduced rate relative to that observed for other antifungals, though it will depend on how the antimicrobial peptide is administered. Besides, although a lower target concentration is required, the absence or change in the specific fungal target through spontaneous mutation can naturally lead to resistance. However, such modification of conserved molecules could, in turn, result in reduced pathogen virulence. In addition, combination therapy, involving the use of AMPs with antibiotics or with other peptides, will likely reduce the development of resistance markedly (Matejuk et al., 2010; Rautenbach et al., 2016).

## Production of AFPs

Despite progress relating to the discovery and characterization of AMPs, their application remains challenging (Wimley and Hristova, 2011). There is a need for effective AMP production in sufficient amounts and purity to more extensively investigate their structure–function relationships, efficacy and safety, especially in clinical treatments. Efficient production is also required to serve market requirements should these investigations be successfully completed. There are three major strategies that one could employ to achieve this, i.e., direct isolation from natural producers, heterologous expression or chemical synthesis.

### Natural Production

Currently, there are not many AMPs that are produced from their natural sources for clinical use. The purification of peptides from natural sources is laborious and expensive due to their low abundance and the multiplicity of compounds present in those sources (Vriens et al., 2014). Because of this, most industrial-scale productions of AMPs are performed by heterologous expression or chemical synthesis. Brief descriptions of two approaches for industrial-scale AMP production from natural sources are presented here: microbial fermentation and proteolysis of food proteins.

Echinocandins are exceptional examples of AMPs produced by microbial fermentation, and further chemical modification in case of the semisynthetic variants. Industrial-scale production of echinocandins is based on fermentation since they possess a high-complexity chemical structure. However, little information has been published about these processes. Echinocandin B, pneumocandin B_0_ and FR901379 are the natural echinocandins produced for commercial purposes from *A. rugulosus, Glarea lozoyensis* and *Coleophoma empetri*, respectively. The optimization of the fermentation process is essential to obtain a competitive product, since the fermentation and purification costs of natural echinocandins are the main variables that influence the overall production costs of semisynthetic derivatives. The clinical applications of natural echinocandins is limited by certain undesirable properties, such as a strong hemolytic activity, as discussed previously (Emri et al., 2013).

Industrial scale AMPs can also be obtained by proteolysis of food proteins and the release of encrypted peptides. Agyei and Danquah (2011) offered a brief description of the process for manufacturing pharmaceutical-grade peptides by this approach. The process involves, firstly, the acquisition of raw materials: food protein and proteolytic enzymes/or microorganisms. By-products from dairy, fish and meat industries are suitable cheap sources for proteins (Sibel Akalin, 2014; Ryder et al., 2016). The second step involves protein hydrolysis. The use of enzymatic hydrolysis is preferred over *in situ* microbial fermentation, particularly in food and pharmaceutical industries due to the lower or absent output of organic solvents and toxic chemical in the process and product (Sibel Akalin, 2014; Ryder et al., 2016). Under industrial-scale conditions, the use of immobilized enzymes offers several advantages over the conventional soluble enzymes, such as milder and controlled conditions and recycling of enzymes used (Sewczyk et al., 2018). The final step is fractionation and isolation of the bioactive peptides. Ultrafiltration, precipitation with solvents and liquid chromatography techniques (e.g., ion exchange, gel filtration) have been proposed for purification of peptides, however, their current implicit high costs make them prohibitive for large scale applications. It is estimated that up to 70% of the capital and operating costs in industrial biotechnology processes may correspond to the separation and purification stages (Brady et al., 2008). Electro-membrane filtration (EMF) is being established as an alternative method for the purification of bioactive peptides. It combines electrophoresis with conventional membrane filtration, being more cost-effective than chromatographic techniques (Bazinet and Firdaous, 2013). AMPs with antifungal activity, such as casocidin-I, kapacin A and lactoferrin-derived peptides can be produced by these methods as well.

### Recombinant Production of AFPs

Considering the often low amounts of AMPs obtained by purification from natural sources and the high costs and difficulties that may arise from chemical synthesis (Li et al., 2010; Hou et al., 2017), recombinant production of AMPs provides a solid option to make these peptides accessible at low cost and high efficiency.

Genetically modified microorganisms facilitate the production and functional expression of any bioactive molecule but, importantly, also allow the production of bioengineered and encrypted peptides that would not be achievable otherwise (Wibowo and Zhao, 2019). Moreover, new sequencing technologies have made available a vast amount of genomic, transcriptomic and metabolic data, providing the means to further explore known and novel AMPs and the rational design of new antifungal peptides (Amaral et al., 2012; Porto et al., 2012; Tracanna et al., 2017).

However, the success of recombinant production can be highly variable. Understanding the composition and physicochemical properties of AMPs influences the selection and design of hosts and expression system. The choice of host, codon bias, protein expression vector, number of copies of the plasmid and fusion proteins can influence the correct synthesis, folding, and secretion of the recombinant peptide through the cell machinery (Deng et al., 2017). There are two other aspects to bear in mind: the toxicity of the AMP for the host and the high instability and susceptibility of peptides to degradation by proteases. To overcome this, AMPs can be synthesized in a form that is fused to another protein (fusion proteins) or in inactive forms (Kosobokova et al., 2016) initially.

*Escherichia coli*, yeasts (mainly *Pichia pastoris*) and plants are the most common recombinant expression platforms for biopharmaceutical proteins. Comprehensive reviews have previously reviewed the different heterologous production platforms available for AMPs (Sanchez-Garcia et al., 2016; Deng et al., 2017). Most AFPs produced by recombinant platforms target plant phytopathogens rather than human fungal pathogens. However, many of these AFPs may represent underutilized resources whose antifungal activity against human fungal pathogens is waiting to be discovered. In the next subsection, a brief overview of the production of AFPs in different hosts with clinical applications is provided.

#### Production of AFPs in Bacteria

*E. coli* is by far the bacterial species that is used most widely as a host for heterologous production of peptides and proteins (Li, 2011). Its genetic configuration is well-known, it is easy to manipulate and there is a broad variety of protein expression vectors and host strains available. The pET vectors (Novagen) are the most commonly used. Among the expression strains, the most popular are *E. coli* BL21 (DE3), deficient in proteases that may lead to protein degradation, or pLysS Origami and Rosetta and C41 (DE3) (Novagen), employed when disulfide bond formation is needed (Rosano and Ceccarelli, 2014). Many *E. coli* strains are unable to export proteins across their outer membrane, and proteins are secreted into the cytoplasm or periplasm generating inclusion bodies (Singh et al., 2015). Therefore, AFPs produced by *E. coli* are usually purified by sonication methods followed by reversed-phase chromatographies, giving relatively low yields. Several examples of AFPs production in *E. coli* are shown in Table 3.

**Table 3:** Expression of antifungal peptides in *E. coli*.

**Type of AFP**	**Name**	**Source**	**Host**	**Expression vector**	**Fusion partner**	**Antifungal spectrum**	**Yield**	**References**
Defensin	PvD1	*Phaseolus vulgaris* L. (common bean)	*Escherichia coli* Rosetta Gami DE3	pET-32 EK/LIC	Thioredoxin	*Candida albicans*	Unknown	de O Mello et al., 2014
Hybrid peptide (lactoferricin+cecropin)	LF15-CA8	*Hyalophora cecropia* (giant silk moth) Bovine lactoferricin (encrypted peptide)	*E. coli* BL21 (DE3)	pGEX-4T-2	GST	Lactoferricin: *C. tropicalis, C. krusei, C. albicans, C. glabrata, Aspergillus* spp*., Cryptococcus* spp.	10 mg/mL	Feng et al., 2014; Fernandes and Carter, 2017
Lactoferricin	Lactoferricin B	Mammals	*E. coli* BL21 (DE3)	pET21d	MMIS	Lactoferricin:*C. tropicalis, C. krusei, C. albicans, C. glabrata, Aspergillus* spp*., Cryptococcus* spp.	Unkown	Kim et al., 2006; Fernandes and Carter, 2017
Cecropin	CeA	*Hyalophora cecropia* (giant silk moth)	*E. coli* BL21 (DE3)	pET-30a	ELK16 self-assembly peptide (GyrA intein)	*C. tropicalis, C. krusei, C. albicans, C. glabrata, Aspergillus* spp*., Cryptococcus* spp.	6.2 mg/mg wet cell	Wang et al., 2018
Peptidyl nucleoside antibiotics	Nikkomycin	*Streptomyces ansochromogenes*	*E. coli* BL21 (DE3)	pET23b	His tag	*C. albicans*	800 mg/L	Li et al., 2005
Echinocandins	PH HtyE	*A. pachycristatus*	*E. coli* BL21 Gold	pET-28b(+)	His tag	*Candida* spp., *Aspergillus* spp.	75 mg/L	Mattay et al., 2018
Magainins	Magainin-2	*Xenopus laevis* (African frog)	*E. coli* BL21 (DE3)	pET-21a	Carbohydrate-binding module, His tag	*C. albicans*, *C. neoformans* *S. cerevisiae*	Unkown	Zasloff, 2002; Ramos et al., 2013
Dermaseptin	Dermaseptin S4	Phyllomedusinae frogs (amphibian skin)	*E. coli* strain BL21 (DE3)	pGEX-4T-1	Glutathione S-transferase (GST)	*C. neoformans* and *A. fumigatus*	Unknown	Belaid and Hani, 2011; Song et al., 2014
Chitin-binding cysteine rich	Tachycitin	horseshoe crab hemocyte (*Argopecten irradians*)	*Escherichia coli* BL21 (DE3)/pLysS	pET-22b()	None	*Paecilomyces variotii, Aspergillus* spp., *F. oxysporum, Neurospora crassa, B. cinerea*, and *Alternaria brassicola*	1 mg/L	Kawabata et al., 1996; Suetake et al., 2002
Defensin	HBD5/ HBD6, HBD26, HBD27	Mammal	*E. coli* BL21 (DE3)	pET-32a (+)	Thioredoxin AHis6	*C. albicans*	Unknown	Huang et al., 2008, 2009

*Bacillus subtilis* has also been explored as host for AFPs production. This includes plectasin (Zhang et al., 2015), cathelicidin (Luan et al., 2014), and the hybrid cecropin A– melittin (Ji et al., 2017) (Table 4). *B. subtilis* is a well-studied species, is non-pathogenic, has been approved by the Food and Drug Administration as a Generally Regarded As Safe (GRAS) microorganism and does not exhibit codon bias. It also reaches high cell density and releases proteins directly to the extracellular medium, simplifying the purification process. However, *B. subtilis* secretes proteolytic enzymes that can degrade the secreted recombinant proteins. Luckily, optimization of the cloning strategies and construction of protease-negative mutants are fostering its wider use (Cui et al., 2018).

**Table 4:** Expression of antifungal peptides in other bacteria and yeasts.

**Type of AFP**	**Name**	**Source**	**Host**	**Expression vector**	**Fusion partner**	**Antifungal spectrum**	**Yield**	**References**
**BACTERIA**								
Cyclic lipopeptide	Iturin A	*Bacillus amyloliquefaciens* and *B. subtilis*	*B. amyloliquefaciens*	Genome shuffling	Protoplast fusion technology	*Saccharomyces cerevisiae*, plant pathogens	172.22 mg/L	Shi et al., 2018
Defensin	Plectasin	*Pseudoplectania nigrella*	*B. subtilis*	pGJ148	Small Ubiquitine-like modifier (SUMO)	*Candida albicans*	5.5 mg/L	Zhang et al., 2015
Polyoxins	Polyoxin P Polyoxin O	*Streptomyces ansochromogenes, S. cacaoi*	*S. ansochromogenes*	pPOL	None	*Alternaria kikuchiana, Aspergillus fumigates, Rhizoctonia solani, Botrytis cinerea* and *Trichoderma viride*	Unknown	Li et al., 2012
Human beta defensin	HBD-1	Humans	*Lactococcus lactis* A164	pOED1	DsbC-Tag	*C. albicans*	Unknown	Choi et al., 2005
**YEASTS**								
*De novo* designed peptide	PAF102	Combinatorial screen against the phytopathogen *Penicillium digitatum*	*Pichia pastoris*	pGAPHA	Plant oleosin	*P. digitatum, Magnaporthe oryzae, Fusarium oxysporum* and *B. cinerea*	180 mg/L	López-García et al., 2015; Popa et al., 2019
Big defensin	AiBD	*Argopecten irradians* (mollusk)	*P. pastoris* GS115	pPIC-9K	None	*C. albicans*	Unknown	Saito et al., 1995; Zhao et al., 2007
Cathelicidin	Protegrin 1 (PG1)	Mammals	*P. pastoris* X-33	pJ912	His tag	*C. albicans*	104 mg/mL	Huynh et al., 2018
Cathelicidin	Protegrin 1 (PG1)	Mammals	*P. pastoris* X-33	pPICZα-A	His tag	*C. albicans*	15.6/100 mL	Niu et al., 2015
Transferrin family	pLF (Porcine lactoferrin)	Sow's milk	*P. pastoris* GS115 (his4)	pPIC9	None	*C. tropicalis, C. krusei*, and *C. albicans*	Unknown	Pecorini et al., 2005; Fernandes and Carter, 2017
Plant defensin	HsAFP1	*Heuchera sanguinea* (coral bells)	*P. pastoris* X-33	pPICZαA	None	*S. cerevisiae, C. albicans*, and *F. culmorum*	40 mg/L	Aerts et al., 2011; Vriens et al., 2015
Radish defensin	RsAFP2	*Raphanus sativus* L. (radish seeds)	*P. pastoris* strain GS115	Unknown	None	*Alternaria* spp*., Fusarium* spp*., Trichoderma* spp.	100 mg/L	Terras et al., 1992; Vriens et al., 2016
Chitinase	VuChiI	*Vigna unguiculata* (L.) (walp.Cow pea)	*P. pastoris* KM71H	pPICZαA	Histag	*P. herquei*	18 mg/L	Landim et al., 2017

Lactic acid bacteria (LAB) have been extensively used for the heterologous production of bacteriocins, antibacterial peptides secreted by bacteria (Rodríguez et al., 2003; García-Fruitós, 2012). Genera such as *Lactobacillus, Leuconostoc, Pediococcus, Lactococcus, Streptococcus*, and *Enterococcus* (König and Fröhlich, 2017) can be part of the human microbiota at different body sites (George et al., 2018) and, for some of these genera, specific strains have been used as probiotics (Harzallah and Belhadj, 2013). However, the production of AFPs in LAB is challenging due to the antimicrobial sensitivity of the host. Choi et al. (2005) attempted the heterologous production of HBD-1 in a nisin Z *L. lactis* producer, but toxicity to the host was apparent (Choi et al., 2005) (Table 4).

In addition to being outstanding secondary metabolite producers, including of antibacterial and antifungal peptides (Harir et al., 2018), in their own right, *Streptomyces* species also offer many potential advantages as hosts for the expression and secretion of proteins (Baltz, 2010). In a small number of cases they have also been used for the heterologous production of AFPs (Li et al., 2012; Roldán-Tapia et al., 2017) (Table 4).

Several fusion partners have been used to facilitate the production of AFPs in bacteria (Li, 2009; Costa et al., 2014). Examples of AFPs produced by *E. coli, Bacillus, Streptomomyces* and *L. lactis* using different fusion partners are shown in Table 4. The His-tag, widely used beyond *E. coli* and consists of a short chain of six histidine residues. The His-tag is often combined with other, fusion tags to improve the production, solubility and recovery of the recombinant protein. Thioredoxin (TRX) and glutathione S-transferase (GST) are ubiquitous enzymes involved in redox and detoxification processes, respectively, which are often used as N-terminal fusion tagsTRX helps with the formation of disulphide bridges of the target protein, especially in strains unable to do so. On the other hand, GST functions as a chaperone that enhances the expression and solubility of recombinant proteins. In addition, GST-tagged fusion proteins can be purified by glutathione affinity chromatography, which facilitates the purification process. MMIS is a modified form of the magainin intervening sequence (MIS) that prevents the antimicrobial activity of the fused peptides until they are released. ELK16 is a self-assembling peptide that induces the formation of cytoplasmic inclusion bodies in *E. coli*. Carbohydrate-binding modules have also been used to enhance purification of AMPs though a cellulose matrix. More innovative fusion partners include protoplast for iturin production (Shi et al., 2018). Overall, fusion partners increase the solubility of the target peptides and protect them from degradation, but there is no evidence of higher yields.

#### Production of AFPs by Yeasts

With respect to heterologous expression, yeasts are fast growing and easy to manipulate genetically. Moreover, they are capable to perform correct protein processing and post-translational modifications. The methylotrophic yeast *Pichia pastoris* has been the preferred yeast for AMP production and for recombinant expression of AFPs (Table 4). *Pichia* protein expression vectors contain the alcohol oxidase gene promoter (AOX 1), inducible by the addition of methanol, which allows the overexpression of the gene introduced downstream. Three strains have been most widely used to produce AFPs: *P. pastoris* X-33, *P. pastoris* GS115, and *P. pastoris* KM71H. The strains differ in their genotypes, which affects the selection of selectable markers, typically antibiotic resistance genes or auxotrophic markers. His-tags are commonly used here also to facilitate the purification of recombinant proteinsaffinity metal-chelating chromatography (Niu et al., 2015; Landim et al., 2017). Plant oleosin fusion technology (Ling, 2007; Bhatla et al., 2010) has also been used for the production of iturin A (Popa et al., 2019). Table 4 shows some examples of AFPs produced by *P. pastoris* for clinical use.

#### Production in Fungi

Filamentous fungi are a well-known source of metabolites and enzymes (Hoffmeister and Keller, 2007), e.g., they naturally produce a wide range of primary metabolites such as organic and fatty acids, and important secondary metabolites, including the antibiotics penicillin, cephalosporin and griseofulvin, or the cholesterol lowering agent lovastatin (Alberti et al., 2017). The attraction of filamentous fungi as hosts for protein recombinant production relates to their relatively inexpensive growing requirements and their ability to naturally secrete large amounts of proteins into the growth medium. They can also perform complex posttranslational modifications including glycosylation, proteolytic cleavage and multiple disulphide bond formation. Moreover, they are very useful when whole synthetic pathways need to be recreated. Species such as *Aureobasidium pullulans, Penicillium chrysogenum*, and *P. digitatum* have been used to produce AFPs*. A. pullulans* has been used to produce several bioactive molecules, such as pullulan, a polysaccharide with numerous applications in health and the food industry, β glucan, and a wide variety of extracellular enzymes. It has also been reported to produce the antibacterial exophilin A, liamocins and heavy oils (Chi et al., 2009). Notably, *A. pullulans* has also been used to produce aureobasidin A as a consequence of homologous recombination (Table 5) (Slightom et al., 2009). *P. chrysogenum* and *P. digitatum* have also been successfully used to produce NFAP2 and AfpB, respectively. *P. chrysogenum* is known to produce a cationic antifungal protein which inhibits zoopathogens and plant-pathogenic fungi. This host has undergone many improvements to optimize fermentation conditions. Finally, *P. digitatum* was originally known as a fruit pathogen but has been used for the homologous production of AfpB.

**Table 5:** Expression of antifungal peptides in fungi and plants.

**Type of AFP**	**Name**	**Source**	**Host**	**Expression vector**	**Fusion partner**	**Antifungal spectrum**	**Yield**	**Reference**
**FUNGI**								
Aureobasidins	Aureobasidin A (BP-1938)	*Aureobasidium pullulans*	*A. pullulans*	pCR2.1 TOPO	None	*Candida* spp.	Unknown	Slightom et al., 2009
Cysteine rich	*Neosartorya fischeri* antifungal protein 2 (NFAP2)	*N. fischeri*	*Penicillium chrysogenum* Q176 GRAS-FDA	pSK275nfap	None	*Candida* spp.	15 mg/L	Tóth et al., 2018
Cysteine rich	AfpB	*P. digitatum* CECT 20796 (PHI26)	*P. digitatum* *P. pastoris* X-33	pBHt2 pPICZαA	None	*Saccharomyces cerevisiae*, *P. italicum*, *P. expansum*, *Botrytis cinerea*, *Magnaporthe oryzae*, *Fusarium oxysporum*, *P. digitatum*	12–20 mg protein/l (*P. digitatum*) 1.2–1.4 mg/l (*P. pastoris*)	Garrigues et al., 2018
**PLANTS**								
Lactoferrin-derived peptides	Lactoferrin+lactoferrampin chimera	Bovine milk	*Nicotiana tabacum*	pBI121	His tag	*C. tropicalis, C. krusei, C. albicans, C. glabrata, Aspergillus* spp*., Cryptococcus* spp.	4.8 μg/g fresh weight	Chahardoli et al., 2018
Dermaseptin	Dermaseptin B1	Skin glands of the South American hylid frog, *Phyllomedusa bicolor*	*Nicotiana tabacum*	pGSA1285	Tandem repeat of *Cladosporium fulvum* Avr4 effector protein CBD	*Agrobacterium tumefaciens* (PTCC 1654), *Pectobacterium carotovorum* (PTCC 1675), *Pseudomonas aeruginosa* (PTCC 1558), *Xanthomonas campestris* (PTCC 1473), and *Ralstonia solanacearum* (ATCC 11696) bacteria, *Alternaria alternata* (PTCC 5224) and *Pythium* spp.	Unknown	Shams et al., 2019


#### Production in Plants

Plant-based expression systems have been explored as production hosts for recombinant expression of AMPs due to their capacity for large scale production and their cost-effectiveness. Advantages of plants are their capability to perform appropriate glycosylation, folding, and disulphide bond formation of recombinant AMPs. There are different genetic approaches to produce AMPs in plants: using whole plants, tissue specific expression, tissue culture, or transient expression (Holaskova et al., 2015). Nuclear transformation has been the preferred technique for plant-derived therapeutic proteins followed by purification from transgenic plants. The tobacco plant (*Nicotiana tabacum*) is the most commonly used transgenic expression system and strains of the bacterial species *Agrobacterium tumefaciens* are the most popular intermediate hosts (Desai et al., 2010). Setting up a higher plant production platform is more expensive than using bacteria, yeast or fungi. However, once the system is established, it is easier to handle and provides high capacity for scale-up. Moreover, plant-based systems do not generally need control of production. Chahardoli et al. used tobacco whole plants as a platform to produce a lactoferrin and lactoferrampin 34 amino acid chimera. To our knowledge this is the first study to produce an AFP in plants (Table 5) (Chahardoli et al., 2018).

### Chemical Synthesis

Chemical synthesis of peptides can be divided in two types: solid- (SPPS) or liquid (solution) phase peptide synthesis (LPPS). In general terms, LPPS is suitable for large-scale manufacture of short peptides or structures that are not easily prepared by SPPS. SPPS is generally used for lower scales or to provide mechanistic insights about peptides and offers the potential for the creation and production of more cost-effective antifungal therapies (Matejuk et al., 2010). Currently, Fmoc SPPS is the preferred method for peptide chemical synthesis due to the versatility and low cost of very high-quality building blocks. The diversity of synthetic peptides entering clinical trials has increased over the last 13 years, stimulating advances in Fmoc SPPS technologies in response to the growing demand for medicinal chemistry and pharmacology (Behrendt et al., 2016). Several groups have synthesized linear (Tran et al., 2008; Magliani et al., 2011; Konno et al., 2015; Cools et al., 2017; Park et al., 2018) and cyclic peptides (Mosca et al., 2000; Schaaper et al., 2001; Konno et al., 2015; Ng-Choi et al., 2019) using Fmoc SPPS.

The long process of isolation and characterization of new natural AMPs delays their clinical use. In this regard, Fmoc SPPS is at the forefront in the design of therapeutic peptides since it permits the easy alteration of features such as hydrophobicity, polarity, charge, structure, and it may also enhance activity and overcome the limitations of natural peptides (Freitas and Franco, 2016). The rational design of synthetic sequences is a new approach of relevance, and results from optimizing the sequence and chemical characteristics shared by different AMPs (pharmacophoric patron) (Freitas and Franco, 2016). Ideally, an antifungal peptide agent should be short, as mentioned previously. *De novo* peptide design may help reducing production costs, potential toxicity and lability, as well as increasing the *in vivo* activity (Steckbeck et al., 2014).

Unfortunately, Fmoc SPPS is currently far from meeting its potential and still cannot compete with the template-based process of expression for the large-scale demand of therapeutics (Behrendt et al., 2016). However, it should be noted that companies specialized in the large-scale manufacture of peptides are currently being established, claiming productions from multi-10 kg/lot (SPPS) to multi-100 kg/lot (LPPS)^1^. This suggests that the versatility of chemical synthesis is on its way to reach the cost-efficiency and scale of peptide expression.

## Current and Potential Applications in Human Medicine

### Combined Therapy With Other Drugs

A recurrence of disease and establishment of chronic fungal infections may result when antifungal treatments are not sufficiently effective. Thevissen (2016) proposed that more efforts should focus on combination therapy in addition to screening for novel antifungal compounds. Synergy between the combined compounds is the main objective of combination therapy, thereby increasing their activity and diminishing their toxicity on the host. Another option is combining an antimycotic (either currently used or novel AMP) with an enhancer molecule that, for example, weakens one or multiple pathogen tolerance mechanisms, such as biofilms, without direct antifungal activity. Either way, it is essential to demonstrate efficacy and safety of the combination before proceeding to clinical trials.

Limited data from clinical trials are available in this regard. A study performed by Candoni et al. showed for the first time that early mortality of patients with invasive aspergillosis was reduced by combined treatment with two antifungal agents (Candoni et al., 2014). In a retrospective study published in 2017, Lee et al. analyzed records from a pediatric department in South Korea and described how the combined therapy of voriconazole and caspofungin was an effective and safe treatment for children with leukemia (Lee et al., 2017). *In vitro* and *in vivo* studies suggests that AFPs are excellent candidates for this type of approaches and have a great potential for clinical success. Lactoferrin-derived peptides such as Lf(1-11) and bLfcin have shown synergy with azole antifungal drugs or amphotericin B, greatly reducing the minimal inhibitory concentrations (MICs) against *C. albicans, C. glabrata, C. krusei, C. parapsilosis, C. tropicalis*, and fluconazole-resistant strains of *C. albicans* (Wakabayashi et al., 1996, 1998; Lupetti et al., 2003; Fernandes and Carter, 2017). Some other examples of these studies are summarized in Table 6.

**Table 6:** Combination of antifungal compounds against pathogenic fungi.

**Compound A**	**Compound B**	**Type of combination**	**Fungus treated**	**Type of study**	**Outcome**	**References**
AmB	bacillomycin D	A - A	*C. albicans*	*in vitro* (keratinocytes)	Synergy; Anti-biofilm and wound-healing activities	Tabbene et al., 2016
	Ds7		*C. tropicalis*	*in vitro*	Synergy; Anti-biofilm and membrane lytic activities	Singh et al., 2017
	Crotalicidin		*Candida* spp.	*in vitro* (HK-2 cells)	Synergy; MIC reduction; less cytotoxic and hemolytic than Amb	Cavalcante et al., 2017
AmB or VCZ	bLfcin		*C. albicans*	*in vitro*	Synergy; 4-16 fold MIC reduction, reduced formation of biofilms	Fernandes and Carter, 2017
FCZ	hLf(1-11)		*C. glabrata, C. krusei, C. parapsilosis, C. tropicalis, C. albicans*	*in vitro*	Synergy; fungicidal effect	Lupetti et al., 2003
	*Ca*Thi		*F. solani*	*in vitro*	Synergy; 100% fungicidal effect	Taveira et al., 2017
Af or Cf	DermaseptinS3(1-16)		*C. glabrata, C. albicans*	*in vitro*	Synergy; MIC reduction	Harris and Coote, 2010
	Renalexin		*C. glabrata, C. albicans*	*in vivo* (mice)	Synergy; MIC reduction; no effect in *in vivo* tests	Harris and Coote, 2010
	Magainin2		*C. glabrata, C. albicans*	*in vitro*	Synergy; MIC reduction	Harris and Coote, 2010
	6752		*C. glabrata, C. albicans*	*in vitro*	Synergy; MIC reduction	Harris and Coote, 2010
	GS14K4		*C. glabrata, C. albicans*	*in vitro*	Synergy; MIC reduction	Harris and Coote, 2010
MUC7 12-mer	Hsn5 12-mer		*C. albicans, C. neoformans*	*in vitro*	Synergy; MIC reduction, low hemolytic activity	Wei and Bobek, 2004
	Amb		*C. albicans, C. neoformans*	*in vitro*	Synergy; MIC reduction	Wei and Bobek, 2004
	Miconazole		*C. albicans, C. neoformans*	*in vitro*	Synergy; MIC reduction	Wei and Bobek, 2004
Cf	Hepcidin 20		*C. glabrata*	*in vitro*	Synergy; MIC reduction	Tavanti et al., 2011
	VCZ		*Pneumocystis jirovecii, Aspergillus* spp.	*in vivo* (children with leukemia)	Overall response 90% after combination treatment; 10% mild liver side effect	Lee et al., 2017
	VCZ		*Candida* spp. *, Aspergillus* spp*., Fusarium* spp.	*in vivo* (humans)	Reduction in early mortality of patients with invasive aspergillosis	Candoni et al., 2014
	LAmB		*Candida* spp*., Aspergillus* spp*., Fusarium* spp.	*in vivo* (humans)	Reduction in early mortality of patients with invasive aspergillosis	Candoni et al., 2014
FCZ	Retigeric acid B (RAB)	A - P	*C. albicans*	*in vitro - in vivo* (mice)	Synergy; inhibition of hyphal formation and adherence to host cells	Chang et al., 2012
	2-adamantanamine (AC17)		*C. albicans*	*in vivo* (guinea pigs)	Synergy; reduction in fungal tissue burden (cutaneous candidiasis)	Lafleur et al., 2013
	FK506		*C. albicans*	*in vivo* (rats)	Synergy; inhibition of biofilm formation in catheter model	Uppuluri et al., 2008
	Cephalosporin A (CsA)		*C. albicans*	*in vivo* (rats)	Synergy; inhibition of biofilm formation in catheter model	Uppuluri et al., 2008
Cf	diclofenac		*C. albicans*	*in vivo* (rats)	Synergy; inhibition of biofilm formation in catheter model	Bink et al., 2012
AmB	bLf peptide 2 - GM-CSF	A - A - P	*C. albicans*	*in vivo* (mice)	Synergy; upregulation of phagocytes; extended survival of mice up	Tanida et al., 2001

*Af, anidulafungin; Cf, caspofungin; FCZ, fluconazole; VCZ, voriconazole; AmB, amphotericin B; MUC7, human mucin-derived peptide; CaThi, Thionine-like peptide from Capsicum anuum; DS7; synthetic peptide derived from Aspergillus giganteous antifungal protein. Hsn5, histatin 5; LAmB, liposomal AmB; hLf(1-11), human lactoferrin peptide 1-11; bLf, bovine lactoferrin; bLfcin, bovine lactoferricin; GM-CSF, granulocyte-macrophage colony-stimulating factor; A, Antifungal; P, Potentiator*.

### Commercial Products and Formulations

P113 is a 12-mer peptide developed by the company PacGen Life Science (Vancouver, Canada) as a mouth rinse formulation for the topical treatment of oral candidiasis. Clinical trials from PacGen demonstrated that oral candidiasis was effectively treated by P113, which compared favorably to the efficacy of nystatin, a standard treatments for oral candidiasis. Vaginal, dermatological and ophthalmic applications are on the list of P113 therapeutic potential (Duncan and O'Neil, 2013). Currently, the P113-containing line of products includes oral rinse solution and spray, feminine soothing spray and cleansing wash, and antibacterial hand cream, among others^2^. NP213/Novexatin was the lead product of NovaBiotics of Aberdeen, UK. It is an arginine-rich cyclic cationic peptide based on human α and β defensins (among others). It was used for treatment of toenails stubborn fungal infections such as onychomycosis (patents PCT/GB2006/004890 and PCT/GB2005/003245). Indeed, independent podiatrist analysis determined the treatment against mild to moderate onychomycosis as being 80% clinically effective. This effectiveness rate is significantly higher than that provided by existing topical treatments for onychomycosis in different territories, including United States and Europe (Duncan and O'Neil, 2013; Fox, 2013)^3^. However, the clinical study did not acomplish the main goal of a Phase IIb study by not showing differences over the placebo treatment under FDA current guidelines^4^.

### Potential Applications

Despite their promising properties, only a few AFPs have reached the clinical phase. One example is hLF(1-11), a peptide that was developed for the systemic treatment of bloodstream and deep tissue infections produced by fungi and bacteria in severely immunocompromised transplant recipients. However, after favorable safety and tolerability clinical trials of hLF(1–11), no more studies have taken place putting on hold the commercialization of this peptide (van der Velden et al., 2009; Martin et al., 2015; Bruni et al., 2016). Another example is CZEN-002, a synthetic octapeptide derived from alpha-Melanocyte-Stimulating Hormone (a-MSH). CZEN-002 modulates immune and inflammatory responses, and has been shown to kill *C. albicans* as well (Fjell et al., 2011; Mahlapuu et al., 2016). This AMP had been in phase II clinical trials for the treatment of vulvovaginal candidiasis. However, no recent development has been reported. It was developed by Zengen, Abiogen Pharma and Lee's pharmaceuticals (Duncan and O'Neil, 2013)^5^.

In addition, there are many cases of peptides with promising properties currently being evaluated pre-clinically (Koo and Seo, 2019). Other pre-clinical examples are described in the review by Duncan and O'Neill (Duncan and O'Neil, 2013) and in the database DRAMP 2.0 (Kang et al., 2019).

### Delivery and Formulations

As discussed, different formulations and delivery strategies for AFPs might be explored depending on the peptide properties, the potential toxicity (suitable for topical and/or systemic use) and the marketing strategies of the companies (e.g., mouth rinse, toothpaste, spray, dermal cream, etc.). Different carriers can enhance the pharmacodynamics and stability, and reduce toxicity of the active peptide, such as liposome encapsulation or the use of peptoids, the D-conformation-based peptide and β-peptides (Sajjan et al., 2001; Porter et al., 2002; da Silva Malheiros et al., 2010; Chongsiriwatana et al., 2011). Diverse pre-clinical delivery tools and formulations are being studied. In one case, Park et al. developed a pH-responsive and redox-sensitive polymer-based AmB-delivery carrier system (Park et al., 2017), by conjugation with histatin 5 acting both as a synergistic antifungal molecule and a targeting ligand against *C. albicans*. Other authors describe that some properties of the peptides could make them suitable for self-delivery systems. Examples include the synthetic killer peptide (KP) and the ultrashort peptide NapFFKK-OH, which, at certain pHs and concentration conditions, undergo a self-assembly process. The results are hydrogel-like aggregates that could slowly release the peptides in physiological conditions, as well as reducing the proteolytic susceptibility and increasing the storage stability of the active compound (Magliani et al., 2011; Albadr et al., 2018). Future clinical studies of these hydrogels and other delivery technologies will determine their safety and efficiency. Additional drug delivery strategies include carbon nanotubes and magnetic nanoparticles (López-Abarrategui et al., 2013; Chaudhari et al., 2016).

## Conclusions

Mycoses are a serious and rising threat to humans. Survival rates remain unacceptably low and no new antifungals have been introduced in more than 13 years since echinochandins and pneumocandins. AFPs have obvious potential as more efficient and safer therapeutic agents than conventional antifungal drugs. Research on AFPs has been highly active and over one thousand peptides have been described. Nevertheless, few molecules have reached late clinical stage studies or have entered the market. Major challenges for AFPs commercialization relate to their specificity and safety. Moreover, stability of the formulations, delivery strategies and the overall therapeutic efficiency together with production costs at industrial scale and regulatory barriers remain to be resolved. The mode of action of AFPs is not fully understood which also raises safety concerns.

Regarding exploitation, AFP production yields from natural sources are very low and requires complex and costly procedures for extraction and purification. Their peptidic nature enables production through recombinant platforms, but scaling-up procedures are not always successful and require extensive optimization. Currently, chemical synthesis is economically viable only for short peptides and high value applications, but novel synthesis and purification technologies such as EMF are on the way to meet these requirements for such short peptides and high value applications.

Notably, indications from the FDA and European regulatory entities indicate that regulatory laws are changing, encouraging companies to invest in antimicrobial discovery and development. Among other measures, new guidance documents have been released, including plainspoken clinical criteria for evaluating antimicrobials and broadening the spectrum of volunteers for clinical trials. Other steps will help in trials designed for the evaluation of drug-resistant pathogens (Fox, 2013).

Considering that the global market for antifungals is set to be worth $12.2 billion^6^, AFPs are commercially attractive candidates in terms of manufacturing costs, options, increasing regulatory acceptance of peptide therapeutics, etc. (Duncan and O'Neil, 2013). Thus, an integral action plan on this field needs to be driven by academy, biotechnological and pharmaceutical companies and regulatory entities in order to enhance and thrust the development of novel antifungal therapies. Nonetheless, the path has been paved for these promising molecules.

## Author Contributions

MF, SA, and EG-G designed and wrote the manuscript. PC critically revised the manuscript. All authors listed have made a substantial, direct and intellectual contribution to the work, and approved it for publication.

### Conflict of Interest

The authors declare that the research was conducted in the absence of any commercial or financial relationships that could be construed as a potential conflict of interest.

## Footnotes

^1^Synthesis—PolyPeptide. Available online at: https://www.polypeptide.com/commercial-manufacturing/ (accessed August 12, 2019).

^2^P113 | Pacgen Life Science. Available online at: http://www.pacgenlife.com/node/171 (accessed August 15, 2019).

^3^Elewski, B. E. *Dermatologists and Podiatrists Interviewed*, 14.

^4^https://www.businesswire.com/news/home/20180928005622/en/Taro-Terminates-Agreement-NovaBiotics

^5^CZEN-002 Available online at: https://www.pharmacodia.com/yaodu/html/v1/chemicals/f84aa65357bec670cbba3ae77711c233.html (accessed August 15, 2019).

^6^http://pharmaceutical-market-research.com
